# Detrimental changes to the health and well-being of healthcare workers in an Australian COVID-19 hospital

**DOI:** 10.1186/s12913-021-07013-y

**Published:** 2021-09-22

**Authors:** Joanne M Stubbs, Helen M Achat, Suzanne Schindeler

**Affiliations:** Epidemiology and Health Analytics, Western Sydney Local Health District, Locked Bag 7118, Parramatta BC, NSW 2124 Australia

**Keywords:** Psychological distress, Mental health, Lifestyle, Pandemic, Retrospective recall, Hospital staff, Repeated measures

## Abstract

**Background:**

Most studies examining the psychological impact of COVID-19 on healthcare workers (HCWs) have assessed well-being during the initial stages or the peak of the first wave of the pandemic. We aimed to measure the impact of COVID-19 and potential changes over time in its impact, on the health and well-being of HCWs in an Australian COVID-19 hospital.

**Methods:**

An online questionnaire assessed current and retrospective physical and mental health; psychological distress (Kessler Psychological Distress Scale); lifestyle behaviours; and demographics, providing measures of health and wellbeing at three phases of the pandemic. Targeted staff were invited to participate via email and in-person. Additional promotional activities were directed to all staff. Changes in general health, mental health and psychological distress were examined using McNemar’s Chi-square. Associations between other categorical variables were tested using Chi-Square or non-parametric equivalents as appropriate. Logistic regression explored risk factors for current distress.

**Results:**

Four hundred thirty-three eligible HCWs answered all (74 %) or part of the questionnaire. Current self-rated health and mental health were significantly better than during the height of the pandemic, but had not returned to pre-pandemic levels. Psychological distress was significantly more common during the height of the pandemic (34.2 %) than currently (22.4 %), and during the height of the pandemic distress was significantly more common among younger than older HCWs. Females were significantly more likely to be distressed that males currently, but not during the height of the pandemic. High distress during the height of the pandemic was more likely to be maintained by HCWs who were less physically active than usual during the height of the pandemic (OR = 5.5); had low self-rated mental health before the pandemic (OR = 4.8); and who had 10 or more years of professional experience (OR = 3.9).

**Conclusions:**

The adverse effects of the pandemic on HCWs have lessened with the easing of pandemic demands, but health and well-being have not reverted to pre-pandemic levels. This indicates continued exposure to elevated levels of stress and/or a sustained effect of earlier exposure. Initiatives that provide ongoing support beyond the pandemic are needed to ensure that HCWs remain physically and mentally healthy and are able to continue their invaluable work.

## Introduction

Preconditions to optimum patient outcomes are the health and safety of healthcare workers (HCWs). The routine work of HCWs exposes them to workplace hazards that go beyond the more common psychosocial and ergonomic factors and include biological, chemical and physical dangers, resulting in this occupational group experiencing among the highest rates of workplace injuries and mental health problems [1, 2]. The COVID-19 pandemic has instigated additional job stressors stemming from workload demands including, but not limited to, longer hours, wearing of personal protection equipment that can be hot and uncomfortable, and a continually changing knowledgebase with accompanying recommendations and procedures [3]. The COVID-19 pandemic has brought to the fore HCWs’ unique responsibilities and vulnerability. HCWs, “every country’s most valuable resource” [4], are integral to ensuring an effective response to the pandemic, which requires that they stay physically and mentally healthy [5].

On the international scale, Australia has had comparatively few cases or deaths – 29,978 cases and 910 deaths as of 17 May 2021 [6]. The first cases in Australia were identified in late January 2020 and peaked at the end of March. Daily case numbers were subsequently low until mid-June 2020, when they started to increase again, peaking in early August 2020. Since late-September 2020, a low number of new cases continues to be reported each day [6]. Case numbers in New South Wales (NSW), where this study was undertaken, followed this national general pattern, but in addition experienced a surge in numbers from mid-December 2020 until mid-January 2021 [7]. Despite lower numbers overall, HCWs in Australia are 2.7 times more likely to be infected than the general public [8].

The psychological impact of COVID-19 on HCWs has been extensively explored. Most published studies have examined well-being during the initial stages or the peak of the first wave of the pandemic. To our knowledge this study is one of the first to measure the effect of COVID-19 on HCWs over time. Some months after the height of the pandemic, HCWs working in an Australian hospital accepting known and suspected COVID-19 positive patients rated their current health and well-being, and also recalled the periods prior to the pandemic and at the height of the pandemic to rate their health, well-being and health behaviours at those times. Our aim was to determine the extent and duration of changes experienced in response to the pandemic – issues that have not yet been explored during this pandemic.

## Method

### Participants

This study was undertaken at a large tertiary teaching hospital that is a designated isolation facility in NSW, Australia. Australia’s first COVID-19 patients were cared for at this hospital, and initially it was the only hospital in NSW to accept COVID-19 patients. The hospital had a testing clinic and dedicated COVID-19 wards, consisting at various times of a ward for suspected COVID cases or patients who were waiting for their test result, a ward for confirmed COVID cases, and a COVID-19 ward within the intensive care unit. Over time other NSW hospitals also accepted COVID-19 positive patients.

We conducted a cross-sectional study of hospital staff, focusing on staff whose primary responsibility was to address the organisation’s response to COVID-19 during the period from mid-March 2020 to the end of May 2020.

### Survey instrument

An online questionnaire was developed using SurveyMonkey to assess: demographic details (age, sex, usual living arrangements, highest educational qualification); physical and mental well-being; lifestyle behaviours (changes in physical activity, smoking, alcohol consumption and sleeping patterns); family and social stressors and their impact on personal well-being; and workplace experiences. Respondents answered a question indicating their informed consent to participate before advancing to the study questions.

Respondents rated their general health before the pandemic, during the height of the pandemic and currently (post-height of the pandemic) as excellent, very good, good, fair, poor, or very poor. Mental health at each of the three time periods was rated on the same scale. Current psychological distress was assessed using the 10 item Kessler Psychological Distress Scale (K10) [9] which asks about the frequency of symptoms of anxiety and depression in the past 4 weeks. In addition, respondents retrospectively answered the questions for how they felt during the height of the pandemic. The order of the current and retrospective versions of the K10 was randomly varied across questionnaires to address any potential order effect on responses. Each item of the K10 is scored from one to five, based on how often the symptom was experienced (none of the time to all of the time). The total scores range from 10 to 50. Data related to family and social stressors and workplace experiences are not examined in this paper.

Contact details for various support services were provided at the end of the questionnaire. The anonymous questionnaire took approximately 15 min to complete and was available from 3 November 2020 to 31 January 2021.

### Recruitment and distribution of study questionnaire

The heads of targeted departments within the hospital, including Emergency (ED), Intensive Care Unit (ICU), COVID-19 testing clinic, COVID-19 wards, Infection Control, Infectious Diseases, Respiratory Medicine, Oral Health, Cardiology, Geriatric Medicine, Ear Nose and Throat (ENT) and General Services, were contacted by the research team to inform them about the study and obtain their support. Multiple modes were used to promote the study and invite participation. An email invitation, with a link to the participant information and consent form and online questionnaire, was sent to staff working in the targeted departments and staff who had a key role in addressing the hospital’s response to COVID-19 (*n =* 1,234). Hard copy versions of the questionnaire and promotional posters including a QR code and web link to the questionnaire were distributed to these departments. Three weeks after the initial email, a reminder was sent. Members of the research team visited the ED, ICU, and COVID clinic to speak to staff directly about the study and distribute flyers with the QR code and web link.

In addition to targeted recruitment, promotional activities directed to all staff were also undertaken. Posters were placed on noticeboards located in the lift areas within the hospital. A promotional table was set-up in common areas (near the food court and at the lifts) on five occasions and flyers were distributed to passing staff. The questionnaire was also available and could be completed either online via an iPad or hardcopy. Articles publicising the study were published in the district’s online staff bulletin, staff social network channel and the hospital’s staff newsletter and included the QR code and web link to the questionnaire. To address potential issues related to English literacy and computer access, researchers visited the General Services department on three occasions to facilitate questionnaire completion by cleaning staff.

### Data analysis

Responses to the general health and mental health questions were converted to dichotomous variables reflecting positive (good, very good or excellent) versus other (fair, poor or very poor) ratings. Total K10 score was calculated by summing the score on each question, provided there was a valid response to at least nine questions. If there were only nine valid responses, the missing score was imputated using the mean of the nine valid scores [10]. Total K10 scores were classified into four categories: low (score 10–15), moderate (16-21), high (22-29) and very high (30 or higher) [11]; high and very high scores indicated psychological distress [10]. Wilcoxon’s Signed Rank test compared current K10 score with that during the height of the pandemic as scores were not normally distributed. McNemar’s Chi-square compared general health, mental health, psychological distress and responses to individual K10 items at different time periods. The relationship between age group and psychological distress was examined using the Cochran-Armitage test for trend, and between years working in professional role and psychological distress was examined using the Mann-Whitney U test. Associations between other categorical variables were tested using Chi-Square. For HCWs who had high or very high psychological distress during the height of the pandemic we explored risk factors for current distress using logistic regression. Variables associated with psychological distress in univariate analyses were added to the model, adjusting for sex; variables significant at *p* < 0.05 were kept in the model. Questionnaires completed in January (*n* = 6) were excluded from analyses examining the effect of month of questionnaire completion.

Data analysis was performed using SAS EG v8.0. The study was approved by the Western Sydney Local Health District’s Human Research Ethics Committee (2020/ETH01674).

## Results

In total, 438 HCWs participated in the study. Response to the email invitation was low (7.9 %), primarily due to the extremely small number of emails that were opened (18.7 %). Most (68.3 %) respondents accessed the questionnaire via the QR code or web link, 22.4 % via the emailed link; the remainder either completed the questionnaire with the assistance of a member of the project team, or a hard copy version in their department or at the promotional table. Anecdotal feedback indicated that some HCWs who had received the email accessed the questionnaire via the QR code rather than the emailed link.

Five HCWs did not fit the eligibility criteria (three were on leave and two were not employed at the hospital during the height of the pandemic) and were excluded from analysis. Approximately one-quarter of HCWs (26.1 %) did not answer all the questions.

Eligible HCWs (*n =* 433) most commonly were female (71.6 %), aged 25–34 years (32.1 %), nurses (39.3 %), working in ICU (20.2 %), in a patient facing role (77.8 %), and in their professional role for a mean of 12.4 years (median 10 years; range 0–42 years) (Table 1).

**Table 1:** Respondent characteristics

Respondent characteristics	n	(%)
**Sex (*n =*** **317)**		
Female	227	(71.6)
Male	89	(28.1)
Other	1	(0.3)
**Age in years (*n =*** **315)**		
< 30	81	(25.7)
30–39	75	(23.8)
40–49	69	(21.9)
50 and over	90	(28.6)
**Usual living arrangement (*n =*** **317)**		
Partner and children	127	(40.1)
Partner and no children	71	(22.4)
Living with parents	43	(13.6)
Single parent	17	(5.4)
Other family arrangement	7	(2.2)
Live alone	32	(10.1)
Shared household or group house or boarder	20	(6.3)
**Highest level of education (*n =*** **318)**		
Left school before completing Year 12	16	(5.0)
Completed Year 12	16	(5.0)
TAFE certificate or diploma	29	(9.1)
Bachelor degree or hospital-based equivalent	115	(36.2)
Postgraduate certificate, diploma or degree	142	(44.7)
**Role**		
Nurse	133	(39.4)
Medical doctor	59	(17.5)
Cleaner	37	(11.0)
Administration/clerical worker	32	(9.5)
Allied Health	26	(7.7)
Oral Health	18	(5.3)
Researcher	11	(3.3)
Other	22	(6.5)
**Area**		
Intensive Care Unit	67	(19.9)
Emergency Department	39	(11.6)
Oral Health	31	(9.2)
COVID ward/clinic	27	(8.0)
Across hospital	17	(5.0)
Respiratory Medicine	17	(5.0)
Allied Health	16	(4.8)
Other	123	(36.5)
**Years working in professional field**		
0–4	83	(28.3)
5–9	56	(19.1)
10+	154	(52.6)
**Ever been told by a doctor or health professional you have:**^a^		
Depression	55	(15.3)
Anxiety	61	(17.0)
Other mental health issues	13	(3.6)
None of these	272	(75.8)

^a^response categories are not mutually exclusive, so sum to > 100 %

### Self-rated general health and mental health

**Fig. 1:**
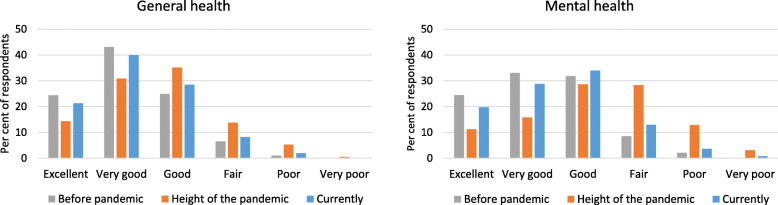
Self-rated general health and mental health before, during and after the height of the pandemic

Most HCWs (89.8 %) stated that their current general health was excellent, very good or good (Fig. 1). Although this proportion was a significant improvement on the 80.4 % who rated their general health during the height of the pandemic as excellent, very good or good (McNemar’s χ^2^ = 27.9, df = 1, *p <* 0.001), it was still significantly lower than before the pandemic (92.5 %; McNemar’s χ^2^ = 5.3, df = 1, *p* = 0.02). A question about personal risk revealed that one in six (16.4 %) had an illness or condition that they believed put them at increased risk during the pandemic.

The majority (82.6 %) of HCWs rated their current mental health as excellent, very good or good, which was markedly more than the proportion who gave the same rating during the height of the pandemic (55.6 %; McNemar’s χ^2^ = 91.7, df = 1, *p <* 0.001), but again was significantly less than the proportion before the pandemic (89.3 %; McNemar’s χ^2^ = 14.1, df = 1, *p <* 0.001) (Fig. 1). In addition to self-report, we also asked about professional input: one-quarter of HCWs had ever been told by a health professional that they had mental health issues; 10 % had more than one mental health issue (Table 1).

Month of questionnaire completion had no effect on general health or mental health ratings at any of the three time periods examined.

### Psychological distress

**Fig. 2:**
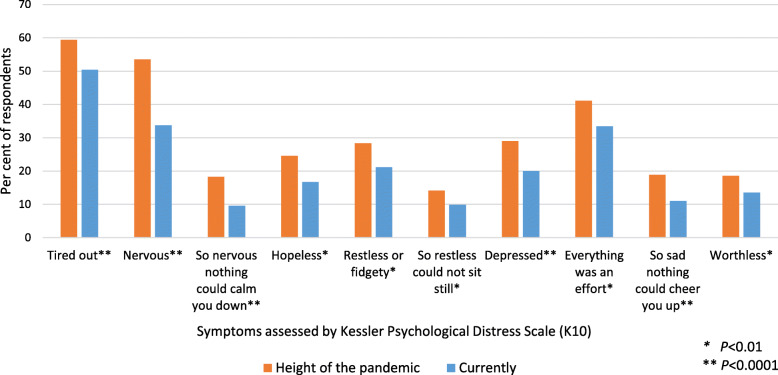
Symptoms experienced ‘some of the time’ or more often, during the height of the pandemic and currently. K10: Kessler Psychological Distress Scale. * *P* < 0.01, ** *P* < 0.0001

For each item of the K10, a significantly higher proportion of HCWs reported that they had experienced the feeling in question ‘some of the time’ or more often during the height of the pandemic compared to the last 4 weeks (currently) (Fig. 2). Feeling ‘tired out’ and ‘nervous’ were frequently reported at both time periods. In terms of absolute change, the difference between the two periods was greatest for feeling ‘nervous’ at least some of the time – decreasing from 54 % at the height of the pandemic to 34 % currently. However, in terms of percentage change, the biggest changes were for ‘so nervous nothing could calm you down’ and ‘so sad that nothing could cheer you up – 48 % and 42 % fewer HCWs, respectively, currently felt this way at least some of the time, compared to during the height of the pandemic.

Mean total K10 score was significantly higher during the height of the pandemic (19.7) than currently (17.4; Wilcoxon Signed Rank test = 10,964, *p <* 0.001). High or very high psychological distress was also significantly more common during the height of the pandemic (34.2 %) than currently (22.4 %; McNemar’s χ^2^ = 25.2, df = 1, *p <* 0.001).

**Fig. 3:**
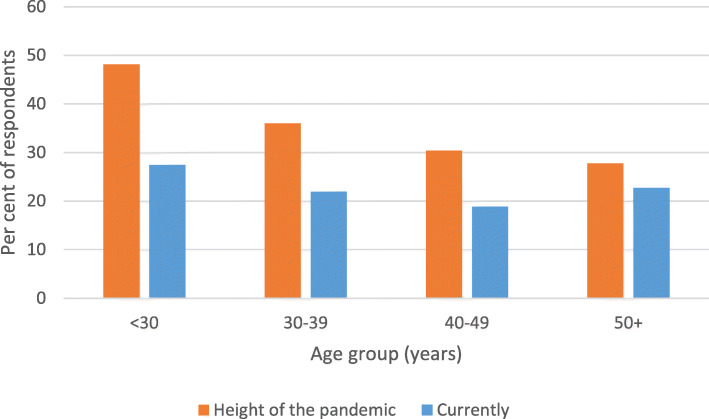
High and very high psychological distress during the height of the pandemic and currently by age

During the height of the pandemic there was no difference between females and males in high or very high psychological distress (38.3 % v 28.1 %, χ^2^ = 2.93, df = 1, *p =* 0.09), but current psychological distress was more common in females (26.0 % v 14.8 %, χ^2^ = 4.52, df = 1, *p =* 0.03). High or very high psychological distress was more common in younger HCWs during the height of the pandemic (Cochran-Armitage trend test Z = 2.83, *p =* 0.005), but there was no effect of age on current distress (Cochran-Armitage trend test Z = 0.80, *p =* 0.43) (Fig. 3). Psychological distress was not significantly associated with years of professional experience either during the height of the pandemic (Mann-Whiney U = 14,074.5, *p* = 0.08) or currently (Mann-Whiney U = 10,171.5, *p* = 0.19). Psychological distress during the height of the pandemic did not vary by month of questionnaire completion (χ^2^ = 1.48, df = 1, *p =* 0.22), but current distress was more common when assessed in November than December (29.8 % v 19.2 %; χ^2^ = 5.11, df = 1, *p =* 0.02).

Since the beginning of the pandemic, almost two-fifths of HCWs had sought assistance to support their well-being or mental health (37.8 %). Although this was primarily informal support from family, friends or others (31.0 %), some sought assistance from a private psychologist, psychiatrist or counsellor (6.8 %), or from the Employee Assistance Program (4.2 %).

### Healthy lifestyle behaviours

During the height of the pandemic, HCWs had less healthy lifestyles than usual as indicated by lower levels of physical activity, increased smoking, and to a lesser extent increased alcohol consumption (Table 2). More than two in every five (42.5 %) HCWs reported sleeping problems; more than a quarter (26.4 %) had difficulty sleeping through the night or waking up during the night for no apparent reason, 15.3 % had difficulty falling asleep, and approximately 10 % experienced early morning waking, or had troublesome dreams or nightmares.

**Table 2:** Lifestyle behaviours during the height of the pandemic

Behaviour	% same as usual	% more than usual	% less than usual
Physical activity (*n =* 354)	25.1	21.8	53.1
Alcohol consumption (*n =* 227)^a^	37.4	38.8	23.8
Smoking (*n =* 39)^a^	33	56	10

^a^35.1 % of HCWs did not drink alcohol; 88.9 % did not smoke

### Maintaining high psychological distress after the height of the pandemic

Univariate logistic regression revealed that physical activity; self-rated mental health before the height of the pandemic; years working in one’s professional field; and working in a high exposure area during the height of the pandemic each individually increased the odds of HCWs who had high psychological distress during the height of the pandemic maintaining high distress later (Table 3). After adjusting for sex and the other variables, the odds that high distress during the height of the pandemic was currently maintained were higher for HCWs who engaged in less physical activity than usual during the height of the pandemic (OR = 5.5); had low self-rated mental health (rated as fair, poor or very poor) before the height of the pandemic (OR = 4.8); and who had worked in their professional field for 10 or more years (OR = 3.9), compared to HCWs with less than 5 years of experience.

**Table 3:** Risk factors for maintaining high psychological distress after the height of the pandemic

Variable	High distress maintained (%)^a^	Logistic regression results				
		Univariate			Adjusted	
		Wald χ2	OR	95% CI	OR	95% CI
Sex						
Female	58		Ref		Ref	
Male	50	0.44	0.7	(0.30-1.82)	0.9	(0.30-2.97)
Age (years)						
18 - 29	53		Ref			
30 - 49	53	0.00	1.0	(0.43-2.41)		
50 +	67	1.18	1.8	(0.62-5.20)		
Poor general health during the height of the pandemic	54	0.07	1.1	(0.51-2.42)		
Poor mental health before the pandemic	76	5.66*	3.4	(1.24-9.16)	4.8	(1.29-18.05)
Increased alcohol consumption	53	0.08	0.9	(0.42-1.90)		
Less physical activity	67	8.07**	3.0	(1.40-6.32)	5.5	(2.02-15.00)
Worked in a high exposure area	45	4.78*	0.4	(0.21-0.92)		
Role						
Medical doctor	47	0.66	0.6	(0.19-1.99)		
Nurse	55	0.18	0.8	(0.38-1.86)		
Other	59		Ref			
Years working in professional field						
0 - 4	42		Ref		Ref	
5 - 9	48	0.16	1.2	(0.43-3.63)	1.2	(0.33-4.06)
10 +	67	4.45*	2.71	(1.07-6.86)	3.9	(1.30-11.56)

^a^% of HCWs with high psychological distress during the height of the pandemic who also had current high distress

^*^p<0.05 , ^**^p<0.01

## Discussion

Research has consistently demonstrated the detrimental effects of COVID-19 on HCWs. The experiences of our HCWs at a designated COVID-19 hospital not only are consistent with that body of evidence in terms of the pandemic’s negative association with their general health, mental health, psychological well-being and health-related behaviours but reveal for the first time the significant lingering changes from pre-pandemic and initial months from its inception, evident several months later. Ongoing psychological distress was related to physical activity during the height of the pandemic, self-rated mental health prior to the pandemic and years of professional experience.

HCWs’ self-reported general health declined during the height of the pandemic, and although it improved some months later, it was not comparable to pre-pandemic levels. Self-reported mental health showed a similar pattern. These findings are consistent with research indicating a relationship between stressful events and adverse physical and psychological health [12], and that the effects can be long lasting as experienced after the severe acute respiratory syndrome (SARS) of 2003 [13].

The proportion of HCWs who reported their current general health to be excellent, very good or good (90 %) was higher than the 84 % of Australians who rated their general health similarly in December 2020 [14]. This may reflect demographic differences between the two samples – employed HCWs might be healthier than the general public which encompasses people from a broader range of ages and physical status [15]. The proportion of HCWs who rated their current mental health as excellent, very good or good was also higher than the Australian general public (83 and 78 %, respectively). This was not unexpected as fewer of our HCWs than the NSW population have ever been told they had depression (15 % v 20 %), anxiety (17 % v 23 %) or any mental health issue (24 % v 30 %) [16].

Comparison of our data with the ABS’s Household Impacts of COVID-19 Survey suggests that during the height of the pandemic, our HCWs were generally more negatively affected by COVID-19 than were members of the Australian general public. The K6 (a subset of the K10 questions) was included in the ABS’s April 2020 telephone survey [17], undertaken during the period we referred to as the height of the pandemic. Responses to our questions about that time were retrospective and were predominately collected online, so although any comparisons should be made with caution, they can provide an indication of differences between HCWs and the general public. Our HCWs were much more likely than the general public to report that at least some of the time during the height of the pandemic they felt nervous (54 % v 35 %), that everything was an effort (41 % v 26 %), hopeless (25 % v 11 %), worthless (19 % v 7 %) and so sad that nothing could cheer them up (19 % v 7 %). These more negative results might reflect HCWs’ increased risk of contracting COVID-19 during the course of their daily work.

Among our HCWs, 35 % met the criteria for high or very high psychological distress during the height of the pandemic, consistent with research in Australia and overseas [18–21] reporting elevated levels of psychological distress amongst HCWs. Significantly fewer of our HCWs were currently distressed (22 %), suggesting that as case numbers and the demands of the pandemic declined, so too did its effect on psychological well-being. However, improved psychological well-being was not equally likely for all HCWs. Decreased physical activity [22] and previous mental health issues [23–25] not only exacerbate the more immediate psychological impact of COVID-19 but also, as indicated by our results, increase the odds of psychological distress several months later. These results are consistent with current knowledge regarding risk factors for psychological well-being. The finding that HCWs with 10 or more years of experience had increased odds of current psychological distress, compared to their colleagues with less than 5 years of working in their professional role, was less expected. Less experienced HCWs have experienced increased psychological distress during the COVID-19 pandemic [21, 26], and higher psychological distress 13–26 months after SARS [13] than their more experienced colleagues. However, our results are more consistent with research reporting an association between experience and increased stress, anxiety and depression [27–29]. Other studies have reported that experience had no effect [26]. These inconsistencies could be related to work and personal circumstances, different assessment methods, and different year groupings, all of which may influence the observed relationship between experience and well-being. After adjusting for other variables, working in a high exposure area was no longer significant which may be related to the stringent guidelines enforced and adhered to by all staff.

Current psychological distress among our HCWs was comparable to that experienced by the Australian general public in November 2020 (21 %) [30]. As a history of mental health problems was less common among HCWs than the Australian general public, it could be expected that potentially fewer HCWs would be predisposed to psychological distress. Instead we found the prevalence of psychological distress in the two samples to be equivalent, which may reflect the ongoing adverse impact of the pandemic on the psychological well-being of HCWs. The similarity in current psychological distress among HCWs and the general public is seemingly inconsistent with self-report which indicated that current mental health was better among HCWs than the general population. This incongruity might be explained by differences in the methods of assessment and precisely what is being assessed, i.e., a single global question asking about one’s mental health and a 10-item instrument assessing various aspects of psychological well-being. Although related, as they do not measure the same constructs global self-rated mental health should not be used as a proxy for psychological distress [31].

During the height of the pandemic, there was no difference between the sexes in high or very high psychological distress. This finding is at odds with most other research which found the psychological impact of COVID-19 to be greater in females than males [21, 24, 26, 32]. Studies in which sex did not show an effect tended to be of nurses and with predominately female participants [26]. Our sample, composed primarily of females (72 %) and nurses (39 %), reflected these characteristics. Usually, a higher level of distress among females is related to their greater exposure to stressors in their daily life [33], but the pandemic changed daily life in many ways. Furthermore, the shared experiences of our HCWs all working in the same COVID-19 hospital with a common purpose, abiding by the hospital’s implementation of strategies and initiatives to keep staff safe, and all experiencing additional social stresses triggered by the pandemic and the consequent restrictions imposed in an effort to curtail its spread, may have negated the commonly reported differences between the sexes. As the height of the pandemic passed, and a degree of normality returned to home, social settings and the workplace, the sex differential was evident in our HCWs. Current distress was significantly higher among female than male HCWs (26 % v 15 %, respectively), with proportions being comparable to that of the Australian general public at a similar time (25 % v 16 %, respectively) [30].

Our results during the height of the pandemic further support the finding that younger age is associated with higher levels of distress amongst HCWs during the COVID-19 and other infectious disease outbreaks [24, 26]. The absence of an effect of age on current distress reflects the dramatic decline in the proportion of our youngest HCWs reporting psychological distress between the height of the pandemic (48 %) and more recently (28 %), which was not evident in older HCWs – especially those aged 50 years and over (28 and 23 %, respectively). Interestingly, although age did not affect the current psychological distress of HCWs, during a similar period (November 2020) distress was more common among younger than older Australians in the general public [30, 34]. Access to information and training about COVID-19 and safe behaviours afforded to HCWs in the hospital environment during the pandemic, but which were less available to the general public, may have alleviated the initial distress experienced by younger HCWs and benefitted their ongoing ability to manage.

COVID-19 was associated with a negative change in our HCWs’ lifestyle behaviours, with more than half being less physically active than usual or smoking more than usual, and almost four in ten consuming more alcohol that usual. These detrimental effects are not unique. Studies of HCWs in Australia [35] and the United States [36] found that approximately half exercised less (44 and 55 %, respectively), and while more than half did not change their level of alcohol consumption, those who did change were mostly drinking more. An Australian study conducted in June 2020, in which 42 % of participants self-identified as a frontline or essential service worker, found that 42 % of ever-smokers increased smoking and 31 % of current drinkers increased their alcohol consumption in the last 4 weeks [24]. The ABS Household Impacts of COVID-19 Survey suggests that changes made by the general public during the pandemic were not as detrimental as those made by HCWs: only 20 % decreased their level of activity and 14 % increased their alcohol consumption [37].

Substance use is a common coping strategy in times of increased stress [36]. The COVID-19 pandemic has exposed HCWs to highly stressful circumstances, putting them under significant psychological pressure which may account for their increased substance use [38]. While a proportion of the population had more leisure time because they were working from home or not working at all [37, 39], anecdotally HCWs were often working longer hours leaving them with less time to be physically active, to connect with family and friends, and for leisure activities in general. Lack of engagement in these activities is particularly concerning as they can effectively enhance mood and moderate stress [40–44]. Although the association between physical activity and mental health during the COVID-19 pandemic has been reported by others [45–47], our results indicate that not only did the majority of HCWs engage in less physical activity than usual during the height of the pandemic, but that this had a sustained detrimental effect on their psychological well-being.

Sleep disturbance amongst HCWs during the COVID-19 pandemic has attracted some attention. Comparable to our 42.5 % of HCWs reporting sleeping problems, a meta-analysis of studies of nurses estimated a 43 % pooled prevalence of sleep disturbance during the COVID-19 pandemic [48]. HCWs in the United States reported a small but statistically significant reduction in total sleep time; more specific aspects of sleep were not measured [36]. Almost one in four (23.6 %) Chinese HCWs reported poor sleep quality, higher than the 18.2 % for all occupational groups combined [49]. The experience of disrupted sleep is not surprising – not only are stressful events known to impair normal sleep, but there is also an association between anxiety, depression and sleep disturbances [50, 51]. The detrimental effect of sleep disturbances on physical health and well-being [52, 53], is a concern at any time, but especially so during a pandemic when HCWs are working longer hours and under greater demands than usual.

At the time of our study, Australia was in the fortunate position that the numbers of COVID-19 cases, hospitalisations and deaths were not as dire as those borne in other countries. Nonetheless, the health and well-being of our HCWs were detrimentally impacted, similar to the experiences of HCWs across the world e.g. [12, 20, 32]. Misinformation and fear of the unknown are world-wide correlates with the COVID-19 pandemic and contribute to psychological distress [54–56]; HCWs in Australia were not immune to the devastation experienced overseas, including the impact on their colleagues, (J.Byrne, personal communication). We postulate that during a global pandemic the adverse effects of working in the healthcare sector are universal and not wholly dependent on local circumstances and severity.

Most studies of the impact of COVID-19 have examined its immediate effects. By including retrospective questions in our cross sectional survey, to collect information about the periods prior to and during the height of the pandemic, we were able to examine potential change over time and sustained impacts. This approach may be subject to errors in recall, which could lead to over- or under-reporting of the impact of the pandemic on HCWs, which should be kept in mind when interpreting the results, although we found no evidence of an effect of time on month of response. We were unable to verify self-reported information with other sources, however this is an accepted issue relevant to most survey-based research.

Response to our email invitation to participate in the study was poor, primarily due to few staff actually opening the email. The additional recruitment methods adopted to increase our sample size not only provided more respondents from our targeted areas but had the added advantage of expanding participation to a broader range of departments within the hospital. This however prevented calculation of a response rate and may have introduced selection bias. Potentially respondents may have been more or less affected by the pandemic than were non-respondents, and consequently our results may over- or under-estimate its effects, which, together with our sample size, may limit the generalisability of our findings to other HCWs. However, as our results are generally consistent with other research examining the immediate and ongoing impact of infectious disease outbreaks, including the current COVID-19 pandemic, on the health and well-being of HCWs we believe that our results may be generalised to other HCWs. The length of time that the questionnaire was available was extended to 3 months to increase our sample size. Although the recall period for retrospective questions varied depending on when the questionnaire was answered, responses did not differ by month of response. We feel confident that recall accuracy was not affected and therefore the validity of results was not compromised by the extended data collection period. The lower prevalence of psychological distress among HCWs responding in December compared to November may indicate progressive recovery from the negative impacts of the pandemic which hopefully will continue over time.

## Conclusions

COVID-19’s impact on the health and well-being of HCWs is evident in countries across the world, even in countries such as Australia where case numbers have been and continue to be comparatively low. Our study is one of the first of its kind for this pandemic, examining changes in the impact of COVID-19 on HCWs over time. HCWs experienced poorer general health and mental health, increased psychological distress, and more frequently engaged in unhealthy behaviours during the height of the pandemic, compared to before its onset and after its first peak. Although self-reported health and well-being had improved some months after the height of the pandemic, neither had returned to pre-pandemic levels, indicating continued exposure to stress and/or an extended effect of exposure. Both possibilities highlight the need for strategies all employers must heed in their duty of care to support HCWs throughout and beyond the pandemic, ensuring HCWs are physically and mentally equipped to continue their invaluable work.

The identification of reduced physical activity, low self-rated mental health and extended years of experience as factors contributing to high psychological distress being maintained can help inform strategies and direct interventions to those HCWs who might be most in need of support.

## Data Availability

The datasets used and/or analysed during the current study are available from the corresponding author on reasonable request.

## Abbreviations

HCW: Healthcare worker.
K10: Kessler Psychological Distress Scale.
